# Toxoplasmose congénitale

**DOI:** 10.11604/pamj.2014.17.260.4200

**Published:** 2014-04-10

**Authors:** Rajae Derrar, Rajae Daoudi

**Affiliations:** 1 Université Mohammed V souissi, Service d'Ophtalmologie A Hôpital des spécialités, CHU de Rabat, Maroc

**Keywords:** Toxoplasmose congénitale, fond d'oeil, acuité visuelle, congenital toxoplasmosis, fundus, visual acuity

## Image en medicine

Il s'agit d'une patiente âgée de 16 ans ayant consulté tardivement pour son acuité visuelle basse. L'examen trouve une acuité visuelle OD à 10/10, la meilleure acuité corrigée au niveau de l'OG est de 1/10. L'examen à la lampe à fente au niveau de l'OD est normal, l'examen de l'OG note l'absence de signes d'inflammation du segment antérieur, un vitré clair, l'examen du fond d'oeil trouve un foyer de choriorétinite cicatriciel pigmenté maculaire. Devant cet aspect, on demande une sérologie de toxoplasmose et du CMV, on a trouvé un taux élevé d'IgG anti toxoplasma gondii attestant de l'ancienneté de l'infection. La toxoplasmose oculaire est une parasitose liée au protozoaire toxoplasma gondii, elle peut être congénitale ou acquise et représente la Cause la plus fréquente d'inflammation du segment postérieur d'origine infectieuse. Le traitement de première intention est basé sur une association de pyriméthamine et de sulfadiazine auquel on ajoute une corticothérapie afin de diminuer l'oedème péri lésionnel. L'indication thérapeutique est basée sur la localisation de lésions dans les formes actives. Au stade cicatriciel, il n'y a plus de récupération possible, d'où l'intérêt du dépistage chez les femmes enceintes avec traitement pré et post natal en cas de toxoplasmose congénitale étiquetée, et une prophylaxie quand elles sont séronégatives.

**Figure 1:**
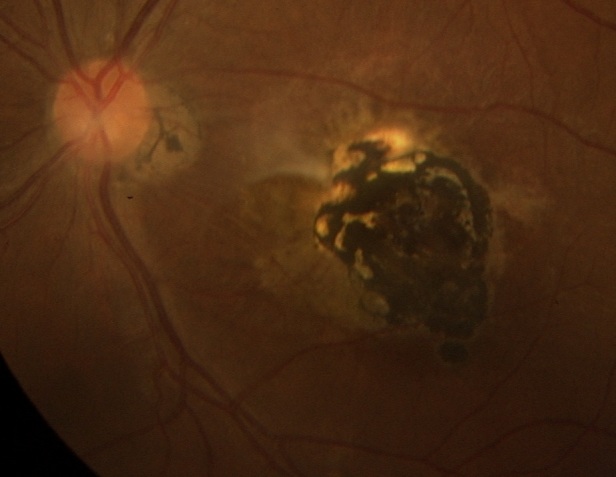
Foyer maculaire cicatriciel pigmenté de toxoplasmose congénitale

